# Cerebral organoids derived from Sandhoff disease-induced pluripotent stem cells exhibit impaired neurodifferentiation[S]

**DOI:** 10.1194/jlr.M081323

**Published:** 2018-01-22

**Authors:** Maria L. Allende, Emily K. Cook, Bridget C. Larman, Adrienne Nugent, Jacqueline M. Brady, Diane Golebiowski, Miguel Sena-Esteves, Cynthia J. Tifft, Richard L. Proia

**Affiliations:** Genetics of Development and Disease Branch, National Institute of Diabetes and Digestive and Kidney Diseases,* National Institutes of Health, Bethesda, MD 20892; National Institutes of Health Undiagnosed Diseases Program, National Institutes of Health Office of Rare Diseases Research and National Human Genome Research Institute,† National Institutes of Health, Bethesda, MD 20892; Department of Neurology and Horae Gene Therapy Center,§ University of Massachusetts Medical School, Worcester, MA 01605

**Keywords:** storage diseases, gangliosides, Tay-Sachs disease, sphingolipids, brain lipids, Clustered Regularly Interspaced Short Palindromic Repeats/Cas9, patient-derived induced pluripotent stem cells, GM2 gangliosidosis, brain development

## Abstract

Sandhoff disease, one of the GM2 gangliosidoses, is a lysosomal storage disorder characterized by the absence of β-hexosaminidase A and B activity and the concomitant lysosomal accumulation of its substrate, GM2 ganglioside. It features catastrophic neurodegeneration and death in early childhood. How the lysosomal accumulation of ganglioside might affect the early development of the nervous system is not understood. Recently, cerebral organoids derived from induced pluripotent stem (iPS) cells have illuminated early developmental events altered by disease processes. To develop an early neurodevelopmental model of Sandhoff disease, we first generated iPS cells from the fibroblasts of an infantile Sandhoff disease patient, then corrected one of the mutant *HEXB* alleles in those iPS cells using CRISPR/Cas9 genome-editing technology, thereby creating isogenic controls. Next, we used the parental Sandhoff disease iPS cells and isogenic *HEXB*-corrected iPS cell clones to generate cerebral organoids that modeled the first trimester of neurodevelopment. The Sandhoff disease organoids, but not the *HEXB*-corrected organoids, accumulated GM2 ganglioside and exhibited increased size and cellular proliferation compared with the *HEXB*-corrected organoids. Whole-transcriptome analysis demonstrated that development was impaired in the Sandhoff disease organoids, suggesting that alterations in neuronal differentiation may occur during early development in the GM2 gangliosidoses.

Sandhoff disease, Tay-Sachs disease, and the GM2 activator deficiency (**Fig. 1A**) are lysosomal storage disorders known as the GM2 gangliosidoses [reviewed in (1)]. They are rare autosomal recessive conditions caused by mutations in the *HEXB* (Sandhoff disease), *HEXA* (Tay-Sachs disease), or *GM2A* (GM2 activator deficiency) genes. The *HEXA* and *HEXB* genes code for the β-hexosaminidase (β-*N*-acetyl-D-hexosaminidase, EC 3.2.1.52) α and β subunits, respectively, which dimerize to form the three isoforms of β-hexosaminidase: A (αβ), B (ββ), and S (αα) (Fig. 1A). The *GM2A* gene encodes the GM2 activator protein, a GM2 ganglioside-binding protein (Fig. 1A). Because β-hexosaminidase A, assisted by the GM2 activator protein, initiates the degradation of GM2 ganglioside through removal of the terminal β-linked *N*-acetyl-galactosamine residue, mutations that inactivate any of these three genes result in lysosomal accumulation of GM2 ganglioside, typically in the form of lamellar membranous inclusions (2).

**Fig. 1.**
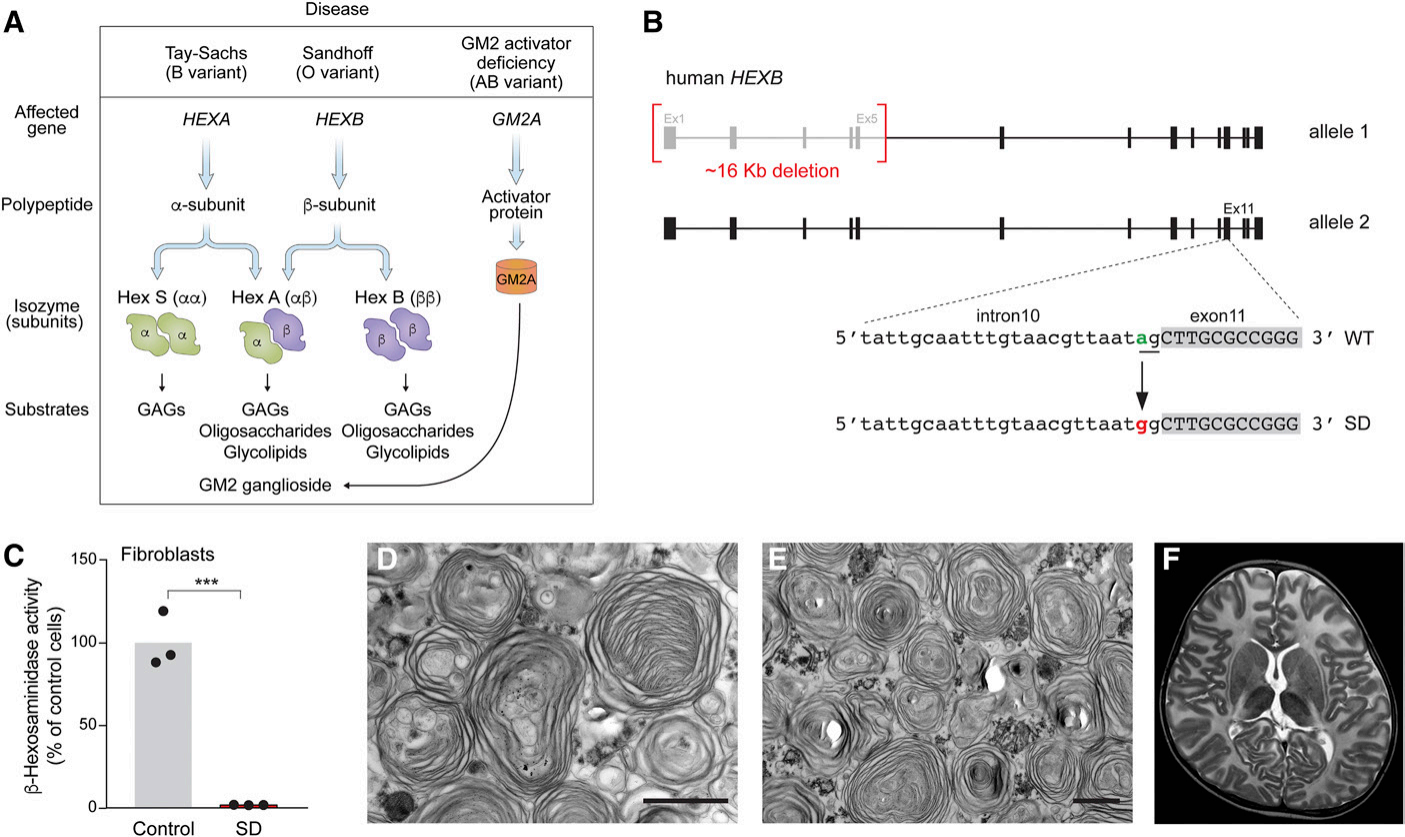
Characterization of the infantile Sandhoff disease patient. A: Diseases, subunits, and substrates associated with GM2 gangliosidoses. B: The infantile Sandhoff disease patient (GSL033) was compound heterozygous for mutations in the *HEXB* gene, with one allele carrying an ∼16 Kb deletion that included the promoter, exons 1–5, and part of intron 5 (top) and the other allele harboring a splice-site point mutation near the 3′ end of intron 10 (bottom). The sequence of the *HEXB* gene shows the single-point mutation (IVS10-2A>G) in the acceptor splice site (underlined) of intron 10. C: β-Hexosaminidase activity detected in lysates from the Sandhoff disease patient’s fibroblasts compared with control fibroblasts. The bars represent β-hexosaminidase activity as percentage of control cells. ***P < 0.001, one-way ANOVA test with Bonferroni correction. D, E: Electron microscopy images of postmortem brain sample of the frontal lobe (D) and thalamus (E). Scale bars, 1 μm. F: MRI image of the patient’s brain at 2 years of age. GAGs, glycosaminoglycans; SD, Sandhoff disease.

The infantile forms of the GM2 gangliosidoses are unremitting neurodegenerative diseases with hypotonia, seizures, macrocephaly, blindness, and progressive loss of motor function and cognition. Onset occurs by 6 months of age, and death ensues typically at 2–5 years (1).

Much of our understanding of the pathogenesis of GM2 gangliosidosis has been uncovered through studies using animal models (3). Sandhoff disease (*Hexb*^−/−^) mice have been the most extensively studied. Like the human infantile patients, the Sandhoff disease mice store abundant GM2 ganglioside in the nervous system, show progressive neurodegeneration, and have a severely shortened lifespan (4). During the disease course, neuroinflammation is prominent, with monocytic infiltration and significant gliosis. Late in the disease, neuronal death occurs by apoptosis (5–7). Whereas the acute neurodegenerative manifestations of the disease are believed to be primarily the result of postnatal processes (8), studies with murine progenitor cells have suggested the possibility that ganglioside storage might also alter neural differentiation (9, 10), raising the possibility that neurodevelopmental defects may also occur in GM2 gangliosidosis patients.

Little is known about early brain development in the GM2 gangliosidoses because of the general inaccessibility of human fetal tissue for study. However, probing human disease progression during development is now possible with the reprogramming of patient-derived somatic cells into induced pluripotent stem (iPS) cells and their further differentiation into cerebral organoids; three-dimensional cell-culture models of early brain development (11, 12). These systems recapitulate the complex cellular behaviors of the developing brain, allowing for the study of fundamental neurodevelopmental mechanisms, such as growth and differentiation, and how they may be altered by disease.

To investigate whether lysosomal β-hexosaminidase deficiency affects early neurodevelopment, we derived iPS cells from the fibroblasts of an infantile Sandhoff disease patient. In addition, we generated isogenic iPS cells in which we corrected the disease-causing *HEXB* mutation using CRISPR/Cas9 genome editing. We then used the Sandhoff disease and control *HEXB*-corrected iPS cells to generate cerebral organoids. We found that the gene expression profile of the cerebral organoids mirrored that observed in fetal neurodevelopment during the first trimester of pregnancy. The Sandhoff disease organoids, which accumulated GM2 ganglioside, exhibited an enlarged size with increased cellular proliferation, and a gene expression pattern indicating impaired differentiation, when compared with isogenic *HEXB*-corrected organoids. Our results suggest that, in addition to its devastating postnatal effects, lysosomal β-hexosaminidase deficiency may alter early neurodevelopmental processes.

## MATERIALS AND METHODS

### Sandhoff disease infantile patient

The Sandhoff disease patient included in this study was enrolled in National Institutes of Health protocol 02-HG-0107, “The Natural History of Patients with Glycosphingolipid Storage Disorders,” with parental consent. All studies involving human subjects were approved by the appropriate review board and abide by the Declaration of Helsinki principles. The patient, a female child of Western European ancestry, was diagnosed by examination of urine oligosaccharide and glycan profiles, and finally by sequencing of the *HEXB* gene from blood leukocyte DNA. During infancy, she developed seizures and neuromuscular weakness and died at 4 years of age. The patient exhibited the characteristic macrocephaly (referring to head circumference size exceeding the 98th percentile value for normal growth for age and gender) that is typical for GM2 gangliosidosis infantile patients (1). Her head circumference measured 55.5 cm at 24 months of age.

### Cell cultures

Human primary fibroblasts were established from a skin biopsy of the patient and were grown in DMEM (Thermo Fisher Scientific, Waltham, MA) supplemented with 10% fetal bovine serum (HyClone Laboratories, GE Healthcare Life Sciences, South Logan, UT). Control fibroblasts were derived from a normal donor skin biopsy.

To generate patient-derived iPS cells, skin fibroblasts from the Sandhoff disease patient were reprogrammed by transfection with episomal vectors encoding the four reprogramming factors, OCT-3/4, SOX2, KLF4, and L-MYC (13) (Applied StemCell, Milpitas, CA). The cloned Sandhoff disease iPS cells had a normal karyotype, exhibited the pluripotent markers characteristic of human stem cells (OCT4, SOX2, and SSEA-4), and were able to form embryoid bodies that differentiated into the three embryonic germ layers. The Sandhoff disease iPS cells were grown in serum-free feeder-free culture conditions on Matrigel-coated (Corning Inc., Corning, NY) wells in mTeSR1 medium (StemCell Technologies, Vancouver, Canada).

Control normal iPS cells were purchased from Alstem (Richmond, CA; catalog number iPS11). They are a footprint-free human iPS cell line derived from normal human foreskin fibroblasts by ectopic expression of *OCT4*, *SOX2*, *KLF4*, and *MYCL* genes using episomal plasmids.

### Creation of isogenic control (*HEXB*-corrected) iPS cells

To create isogenic control iPS cells, we first corrected the intron 10 acceptor splice-site mutation in one of the Sandhoff disease patient’s *HEXB* alleles in the parental Sandhoff disease iPS cells using the CRISPR/Cas9 editing technology (14). We designed a single-guide RNA (sgRNA) containing a 20 bp target sequence corresponding to the 3′ end of intron 10 and the 5′ portion of exon 11 of the human *HEXB* gene, 5′ GTAACGTTAATGGCTTGCGC 3′, which was followed by a protospacer adjacent motif (PAM) sequence, NGG. The sgRNA forward (5′ GTAACGTTAATGGCTTGCGC 3′) and reverse (5′ GCGCAAGCCATTAACGTTAC 3′) sequence oligonucleotides were annealed, phosphorylated, and subcloned into the CRISPR/Cas9 plasmid pSpCas9(BB)-2A-Puro (15) to create the SD/pSpCas9(BB)-2A-Puro plasmid. pSpCas9(BB)-2A-Puro (PX459) was a gift from Feng Zhang (Addgene plasmid #48139; Addgene, Cambridge, MA). To correct the mutant sequence, a single-stranded 181-base repair oligodeoxynucleotide containing a G→A correction for the mutation in one of the patient’s *HEXB* alleles, and 90 bases of homologous sequence flanking each side of the correction were designed: 5′ AAATTATGTTCCTAGTAATAATGCCTTAAACTTTCAATTT­CATCTACTGTTCTAGGCCTAATAATATGTATTGCAATTTGT­AACGTTAATAGCTTGCGCCAGGTACCATAGTTGAAGTATGGAAAGACAGCGCATATCCTGAGGAACTCAGTAGAGTCACA­GCATCTGGCTTCCCTGTAAT 3′. The repair oligodeoxynucleotide also contained a silent point mutation, which would disrupt the *HEXB* target PAM sequence and prevent Cas9 recutting, as well as two additional silent point mutations to create a KpnI restriction enzyme site for screening.

Patient-derived iPS cells were then transfected using the human stem cell Nucleofector Kit 1 (Lonza, Rockville, MD) to deliver 20 μg of the SD/pSpCas9(BB)-2A-Puro plasmid and 100 pmol of the repair oligodeoxynucleotide. After 48 h, transfected iPS cells were treated with puromycin (1 μg/ml) for 48 h. After selection, iPS cells were grown in mTeSR1 until the colonies were large enough for hand-picking. Single colonies were grown individually and expanded for analysis (recovery of β-hexosaminidase activity and sequence confirmation).

For sequencing, the region in the *HEXB* gene containing the acceptor splice-site mutation was amplified by PCR using the flanking primers 5′ CAAACCTAAGGTTGATGAAAC 3′ and 5′ GTTTCATCAACCTTAGGTTTG 3′. The following conditions were used: denaturation 94°C for 1 min, amplification 58°C for 1 min, and extension 72°C for 1 min (40 cycles). The nonedited fragment was 453 bp in length. The PCR fragment was subcloned into the pCR 4-TOPO vector (TOPO TA cloning kit for sequencing; Thermo Fisher Scientific) and sequenced using the T3 primer.

Potential off-target regions for the sgRNA were predicted with the Optimized CRISPR Design website (crispr.mit.edu) (15) and amplified in the *HEXB*-corrected iPS cell clones using the primers listed in supplemental Table S1. The following conditions were used for the five primer sets: denaturation 94°C for 1 min, 55°C for 1 min, and 72°C for 1 min (40 cycles). PCR fragments were sequenced using the corresponding forward primer.

### β-Hexosaminidase assays

iPS cell lysates or human fibroblasts prepared in 0.1 M citric buffer (pH 4.2) containing 0.1% Triton X-100 were assayed for β-hexosaminidase activity with 4-methylumbelliferyl *N*-acetyl-β-D-glucosaminide (Sigma-Aldrich, St. Louis, MO) (16). Values were normalized using β-galactosidase activity and measured using 4-methylumbelliferyl β-D-galactopyranose (Sigma-Aldrich) (17). Activity was calculated as β-hexosaminidase activity normalized by β-galactosidase activity per minute and expressed as percentage of control cells.

### Generation of cerebral organoids from iPS cells

Cerebral organoids were generated using parental Sandhoff disease iPS cells and *HEXB*-corrected iPS cells according to the protocol designed by Lancaster and colleagues (12, 18), with the following modification on embryo body formation. Embryo bodies were generated on AggreWell 400 plates (StemCell Technologies) in AggreWell medium (StemCell Technologies) and maintained in AggreWell medium until neural induction on day 5 of culture. Cerebral organoids were grown in suspension in 125 ml spinner flasks on a low-speed microstirrer (Wheaton, Millville, NJ) at 25 rpm in a 5% CO_2_ tissue-culture incubator for up to 14 weeks, and harvested at indicated time points for analysis.

Organoid size was analyzed by determining perimeter of the individual organoids from digital images taken of the organoids at week 4 and week 10 using ImageJ software (National Institutes of Health).

### Histological analysis of cerebral organoids

Organoids were fixed in 4% paraformaldehyde in PBS for 20 min at 4°C, followed by overnight incubation at 4°C in 30% sucrose in 0.2 M phosphate buffer, pH 7.5. Tissues were embedded in OCT compound (Thermo Fisher Scientific), frozen, and later sectioned for ganglioside immunodetection (19). Frozen sections were warmed to room temperature, air-dried for 10 min, fixed in cold acetone for 10 min at −20°C, and again air-dried. After PBS rehydration, sections were incubated with 5% normal goat serum in PBS for 1 h at room temperature and then incubated with one of the following antibodies for 1 h at room temperature: anti-neuronal β3 tubulin monoclonal antibody clone TUJ1 (mouse IgG, catalog number 801201; BioLegend, San Diego, CA); anti-ganglioside GM2 monoclonal antibody, clone MK1-16 (mouse IgM, catalog number A2576; TCI, Tokyo, Japan) (20); anti-ganglioside GD3, clone R24 (mouse monoclonal IgG3, catalog number ab11779; Abcam, Cambridge, MA); anti-galactosylceramide, clone mGalC (mouse monoclonal IgG3, catalog number MAB342; Sigma-Aldrich); anti-myelin basic protein (rabbit polyclonal IgG, catalog number AB5864; Sigma-Aldrich). After three washes in PBS, sections were incubated for 1 h at room temperature with appropriate secondary antibodies: Alexa Fluor 488-labeled anti-mouse IgM (catalog number A21042; Molecular Probes, Thermo Fisher Scientific), Alexa Fluor 594-labeled anti-mouse IgM (catalog number 21044; Molecular Probes, Thermo Fisher Scientific), or Alexa Fluor 594-labeled anti-mouse IgG (catalog number A11032; Molecular Probes, Thermo Fisher Scientific). Cryosections were mounted for microscopy on glass coverslips using Fluoroshield mounting medium with DNA-binding dye, DAPI (Abcam). Antibody-stained sections were examined using a confocal laser-scanning microscope (LSM 780; Carl Zeiss, Inc., Thornwood, NY) and images were acquired using the Zen 2012 software (Carl Zeiss, Inc.). Average expression of GM2, GD3, galactosylceramide, and myelin basic protein was determined by the quantification of the fluorescence intensity of the anti-GM2 antibody corresponding to an entire organoid section using ImageJ software, normalized with the fluorescence intensity of DAPI for the same section. For cholera toxin B subunit staining, frozen organoid sections were fixed in cold acetone for 10 min at −20°C, air-dried, blocked with 5% normal goat serum in PBS for 1 h at room temperature, and then incubated with FITC-conjugated cholera toxin B subunit (Sigma-Aldrich) for 45 min at room temperature. Fluorescence-stained sections were mounted and examined on a confocal laser-scanning microscope as described above. Cholera toxin B subunit staining fluorescence intensity was quantified as described above.

Cell proliferation within organoid sections was determined by labeling with 5-bromo-2-deoxyuridine (BrdU) (Thermo Fisher Scientific) for 4 h in culture. The cells that incorporated BrdU were detected using Alexa Fluor 488-labeled anti-BrdU antibody (A23210; Molecular Probes, Thermo Fisher Scientific) according to the manufacturer’s protocol. Fluorescently stained sections were mounted and examined on a confocal laser-scanning microscope as described above. Ten fields per organoid section were examined, and the percentage of BrdU^+^ DAPI^+^ nuclei was quantified. Similarly, proliferation of Sandhoff and *HEXB*-corrected iPS cells was determined by incorporation of 5-ethynyl-2′-deoxyuridine (EdU) for 4 h in culture. Cells were fixed in 4% paraformaldehyde in PBS for 20 min at 4°C. EdU-labeled iPS cells were detected using the Click-iT Plus EdU kit (Molecular Probes, Thermo Fisher Scientific) according to the manufacturer’s manual. Fluorescently stained samples were mounted and examined on a confocal laser-scanning microscope as described above. Ten fields per iPS cell type were examined, and the percentage of EdU^+^ DAPI^+^ nuclei was quantified.

Cell apoptosis within organoids was detected by in situ TUNEL on frozen sections using the Apoptag apoptosis detection kit (Sigma-Aldrich) following the manufacturer’s instructions. Cryosections were mounted and examined on a confocal laser-scanning microscope as described above. Ten random fields chosen from an entire organoid section were examined, and the percentage of TUNEL^+^ DAPI^+^ nuclei was quantified.

Transmission electron microscopy of postmortem human brain and cerebral organoids was performed as described (21).

### Adeno-associated virus-mediated expression of β-hexosaminidase in organoids

Using a 5 μl Hamilton syringe equipped with a custom-made 32 gauge small hub needle (10 mm, beveled 25°) (Hamilton, Reno, NV), Sandhoff disease organoids at week 4 were injected with 1 μl of adeno-associated virus (AAV) (2 × 10^9^ vg) carrying a 1:1 ratio of AAVrh8 vectors encoding cynomolgus macaque *HEXA* and *HEXB* subunits (AAV-*HEXA/B*) (22) or AAVrh8-GFP (2 × 10^9^ vg) as a control virus. Organoids were harvested 11 days later for analysis of size, β-hexosaminidase activity, and GM2 ganglioside content.

### RNA-sequencing and bioinformatics analyses

Sandhoff disease and *HEXB*-corrected cerebral organoids were harvested at weeks 8 and 10 of culture and RNA was extracted (four samples at each time point, each consisting of four to six organoids) using the RNeasy Mini kit (Qiagen, Hilden, Germany). RNA was quantified using the Agilent RNA 6000 Nano kit (Agilent Technologies, Santa Clara, CA) on a BioAnalyzer 2100 (Agilent Technologies). RNA (1 μg, RIN >8) was used to prepare RNA-sequencing (RNA-Seq) libraries with the TruSeq Stranded mRNA Library Prep kit (Illumina, San Diego, CA) according to the manufacturer’s protocol. Library DNA concentrations were measured using the Quant-iT PicoGreen dsDNA assay kit (Thermo Fisher Scientific). All samples were normalized according to concentration and pooled. Single-end 50 bp sequencing was performed on an Illumina HiSeq 2500. Reads were mapped to the human hg19 reference genome using the ELAND aligner (Illumina). Reads per kilobase of transcript per million (RPKM) values were determined using the Genomatix Software Suite (Genomatix, Munich, Germany).

Whole transcriptomes from Sandhoff disease and *HEXB*-corrected organoids at weeks 8 and 10 of culture were compared with RNA-Seq gene expression data from 16 normal human dorsolateral prefrontal cortex specimens at the fetal and infancy developmental stages (obtained from the Allen Brain Atlas; www.brain-map.org) (23). Pearson correlation analysis was computed between the gene expression levels, quantified as RPKM, of the four Sandhoff disease and four *HEXB*-corrected samples for each time point and the values for the 16 control human dorsolateral prefrontal cortex samples (23, 24). The National Center for Biotechnology Information Gene Expression Omnibus (GEO) accession number for the RNA-Seq data is GSE106311.

Genes that were expressed at levels RPKM >3 in both cerebral organoids and brain samples were included for Pearson correlation analysis. Heat maps were generated using Prism 7 software (GraphPad, La Jolla, CA).

Differential gene expression analysis was performed by the DESeq2 algorithm using Genomatix Software Suite. A threshold for the log2 (fold change) of expression/enrichment level (*HEXB*-corrected vs. Sandhoff disease) of 1.5 and an adjusted *P*-value threshold ≤0.05 (Wald test) were used for the analysis.

Gene ontology analysis was performed using Genomatix Pathway System (Genomatix Software Suite) through the analysis of the top 100 upregulated and the top 100 downregulated genes for *HEXB*-corrected organoids compared with Sandhoff disease organoids at week 10 of culture. The biological processes category was plotted selecting the top 14 terms ranked by *P* value.

### Statistical analyses

Statistical significance was calculated using the Student *t*-test or one-way ANOVA with Bonferroni correction. *P* ≤ 0.05 was considered statistically significant.

## RESULTS

### Creation of isogenic control (*HEXB*-corrected) iPS cells from Sandhoff disease iPS cells

The infantile Sandhoff disease patient was identified as compound heterozygous for *HEXB* mutations. One of the *HEXB* alleles carried the common ∼16 Kb deletion, which includes the *HEXB* promoter, exons 1–5, and part of intron 5, previously shown to result in undetectable HEXB mRNA (Fig. 1B) (25, 26). The other allele was found to carry a novel point mutation in the intron 10 acceptor splice site (IVS10-2A>G) (Fig. 1B). As expected by the severity of the mutations found on each allele of the gene, fibroblasts from the patient showed nearly absent β-hexosaminidase activity (Fig. 1C).

Electron microscopy analysis of frontal lobe and thalamus samples obtained from the postmortem brain of the patient showed the presence of abundant multilamellar bodies in the cytoplasm of brain cells, indicating the accumulation of lipid consistent with the Sandhoff disease diagnosis (1) (Fig. 1D, E).

The patient also exhibited the characteristic macrocephaly seen in most GM2 gangliosidosis infantile patients. MRI examination of the brain of the patient found no ventricular involvement, consistent with megalencephaly (Fig. 1F).

**Fig. 2.**
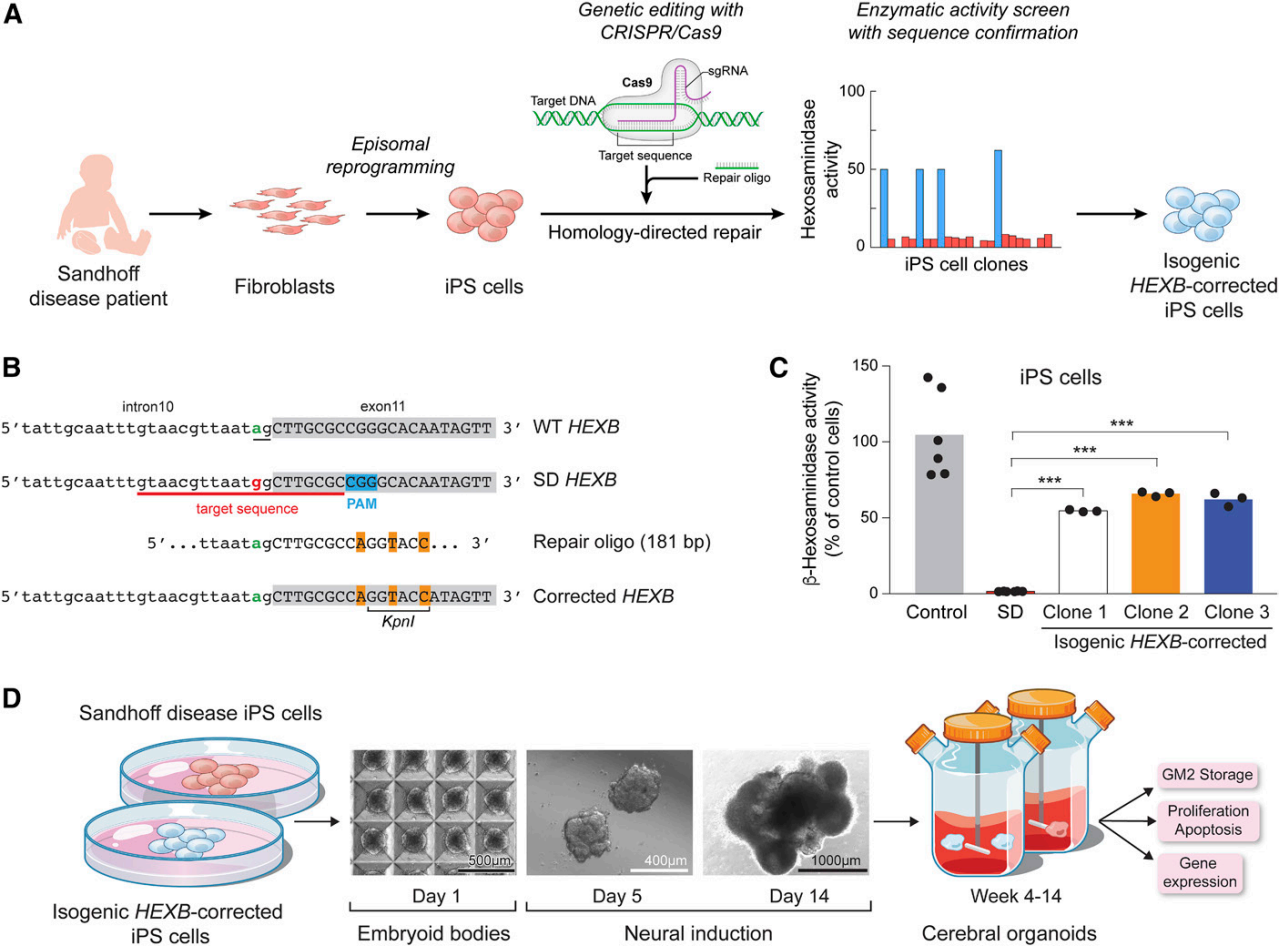
Creation of isogenic control (*HEXB*-corrected) iPS cells and generation of cerebral organoids. A: Strategy for mutation correction to create isogenic control iPS cells. Fibroblasts from the Sandhoff disease patient, GSL033, were transfected using episomal vectors expressing reprogramming factors to generate an iPS cell line. Sandhoff disease iPS cells were transfected with a CRISPR/Cas9 vector expressing the *HEXB*-targeted sgRNA together with a single-stranded repair oligodeoxynucleotide (oligo) to correct the splice-site point mutation through homology-directed repair. iPS cell clones were screened for β-hexosaminidase activity to identify those recovering about 50% of the enzymatic activity found in the control iPS cell line and sequenced to confirm editing. B: Sequence of the targeted region of the *HEXB* gene. WT sequence, mutant SD sequence (showing the 20 bp sgRNA target sequence and the PAM sequence), a segment of the repair oligodeoxynucleotide, and the corrected *HEXB* gene (carrying the correct base in the acceptor splice site of intron 10, underlined) are shown. Sequences corresponding to exon 11 are shadowed in gray. The silent mutations to disrupt the PAM sequence and to create a *Kpn*I site are highlighted in orange. C: β-Hexosaminidase activity of isogenic *HEXB*-corrected iPS cell clones. CRISPR/Cas9-edited iPS cell clones were isolated and screened for the recovery of about 50% of β-hexosaminidase activity in vitro. Three *HEXB*-corrected isogenic iPS cell clones (*HEXB*-corrected clones 1, 2, and 3) were further analyzed. β-Hexosaminidase activity was normalized by β-galactosidase activity per minute. Bars represent the mean β-hexosaminidase activity as percentage of control iPS cells. ***P < 0.001, one-way ANOVA test with Bonferroni correction between SD and each corrected clone. D: General scheme for the generation of cerebral organoids. Cerebral organoids were generated using the parental Sandhoff disease and the three isogenic *HEXB*-corrected iPS cell clones. This method leads to a rapid development of brain-like tissue as a cerebral organoid. Organoids were grown for up to 14 weeks and were analyzed for GM2 ganglioside accumulation, cell proliferation and apoptosis, and gene expression. SD, Sandhoff disease.

We generated iPS cells from skin fibroblasts established from the patient (**Fig. 2A**) (13). As expected, Sandhoff disease iPS cells showed greatly reduced total β-hexosaminidase activity compared with a control iPS cell line (Fig. 2C).

To create isogenic control iPS cells, we edited the genome of the Sandhoff disease iPS cells to correct the *HEXB* acceptor splice-site mutation (Fig. 2A, B). We designed a sgRNA with a 20 bp target sequence corresponding to the 3′ end of intron 10 and the 5′ portion of exon 11 of the human *HEXB* gene to produce a Cas9-induced double-strand break approximately 6 bp from the acceptor splice-site mutation on the *HEXB* allele. A repair oligodeoxynucleotide, which contained a G→A correction for the splice-site mutation, was used as a template for the homology-directed repair process induced by Cas9 cleavage (Fig. 2B). The patient-derived iPS cells were transfected with a plasmid expressing Cas9 and the sgRNA, and with the repair oligodeoxynucleotide. Puromycin-selected iPS cell clones were isolated and tested for β-hexosaminidase activity (Fig. 2A). This approach allowed for rapid screening of the iPS cell clones to identify those in which the *HEXB* gene had been functionally corrected. Thirty-eight individual clones were screened in this manner, and four that expressed elevated β-hexosaminidase activity were further analyzed by sequencing. Three of these selected clones were found to have correct editing of the *HEXB* gene based on the repair oligodeoxynucleotide sequence and were named isogenic *HEXB*-corrected clones 1, 2, and 3. The three clones expressed about half of the total β-hexosaminidase activity detected in normal iPS cells, consistent with the correction of one of the mutated *HEXB* alleles (Fig. 2C).

Potential off-target loci for the sgRNA were predicted by the Optimized CRISPR Design website (15). The top five off-target loci were sequenced in the three isogenic *HEXB*-corrected clones and none were found to be modified (supplemental Table S2).

### Isogenic *HEXB*-corrected cerebral organoids lack GM2 storage

Cerebral organoids generated using iPS cells form complex brain-like structures in suspension culture, and have been used to model normal human brain development and disease (11, 12, 23, 27, 28). To determine whether β-hexosaminidase deficiency affects early neurodevelopment, we generated cerebral organoids from Sandhoff disease iPS cells and isogenic *HEXB*-corrected iPS cells (Fig. 2D). The organoids were grown in suspension culture for up to 14 weeks and then analyzed for GM2 ganglioside accumulation, cell proliferation and apoptosis, and gene expression (Fig. 2D).

**Fig. 3.**
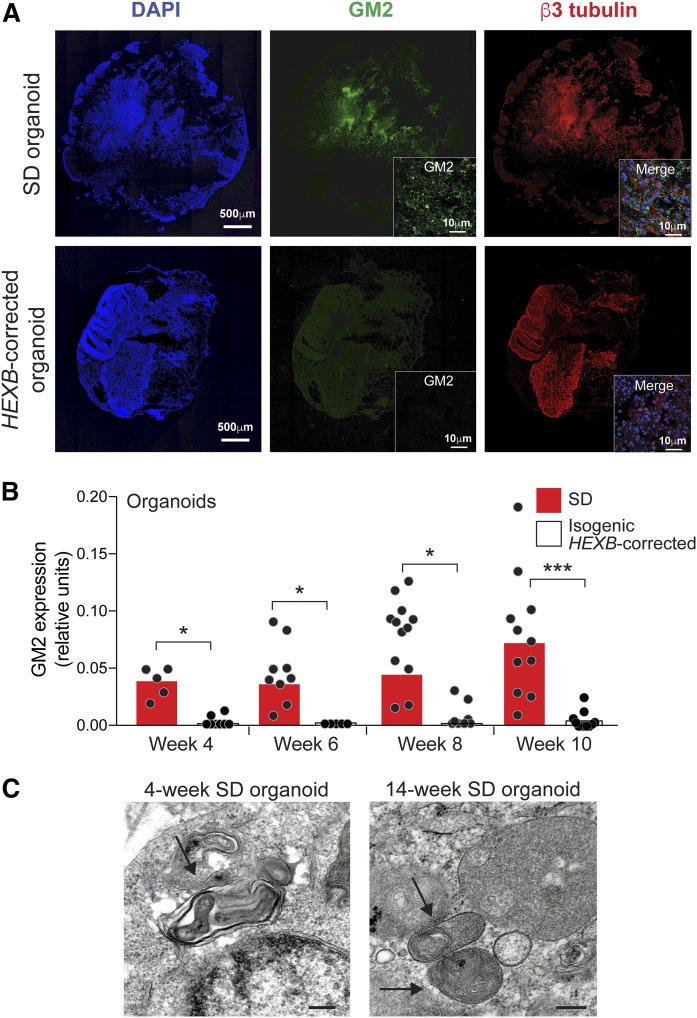
Sandhoff disease cerebral organoids accumulate GM2 ganglioside. A: Frozen sections of Sandhoff disease (SD; top panels) and isogenic *HEXB*-corrected organoids (bottom panels) at week 7 of culture were stained with DAPI (left), anti-GM2 ganglioside (center), and anti-β3 tubulin (right). Representative images of entire organoid sections are shown. The insets show higher magnification views of GM2 staining (center) and DAPI, GM2 and β3 tubulin merged staining (right). B: Quantification of GM2 ganglioside expression in SD organoids (red bars) and isogenic *HEXB*-corrected organoids (white bars) from week 4 up to week 10 of culture. GM2 expression was calculated as anti-GM2 ganglioside antibody fluorescence intensity normalized by DAPI intensity quantified by ImageJ software. Bars represent mean GM2 expression and each dot represents the value corresponding to an entire organoid section, with two sections per organoid. *P < 0.05, ***P < 0.001, *t*-test analysis between SD and the corrected clone at each time point. C: Electron microscopy of SD organoids after week 4 and week 14 of culture showing multilamellar bodies (arrows). Scale bar, 200 nm.

A hallmark of Sandhoff disease is the accumulation of GM2 ganglioside (3, 29). We examined the level of GM2 storage by immunostaining Sandhoff disease cerebral organoid frozen sections, and found the presence of GM2 ganglioside as early as at week 4 of culture, mainly in cells located in areas positive for neuronal β3 tubulin expression (**Fig. 3A**). In contrast, isogenic *HEXB*-corrected organoids accumulated significantly less GM2 ganglioside (Fig. 3A, B). Electron microscopy of Sandhoff disease organoids at week 4 and week 14 of culture showed the presence of inclusion bodies (Fig. 3C), similar to the structures found in the Sandhoff disease patient’s brain (Fig. 1D, E), although not as abundant.

**Fig. 4.**
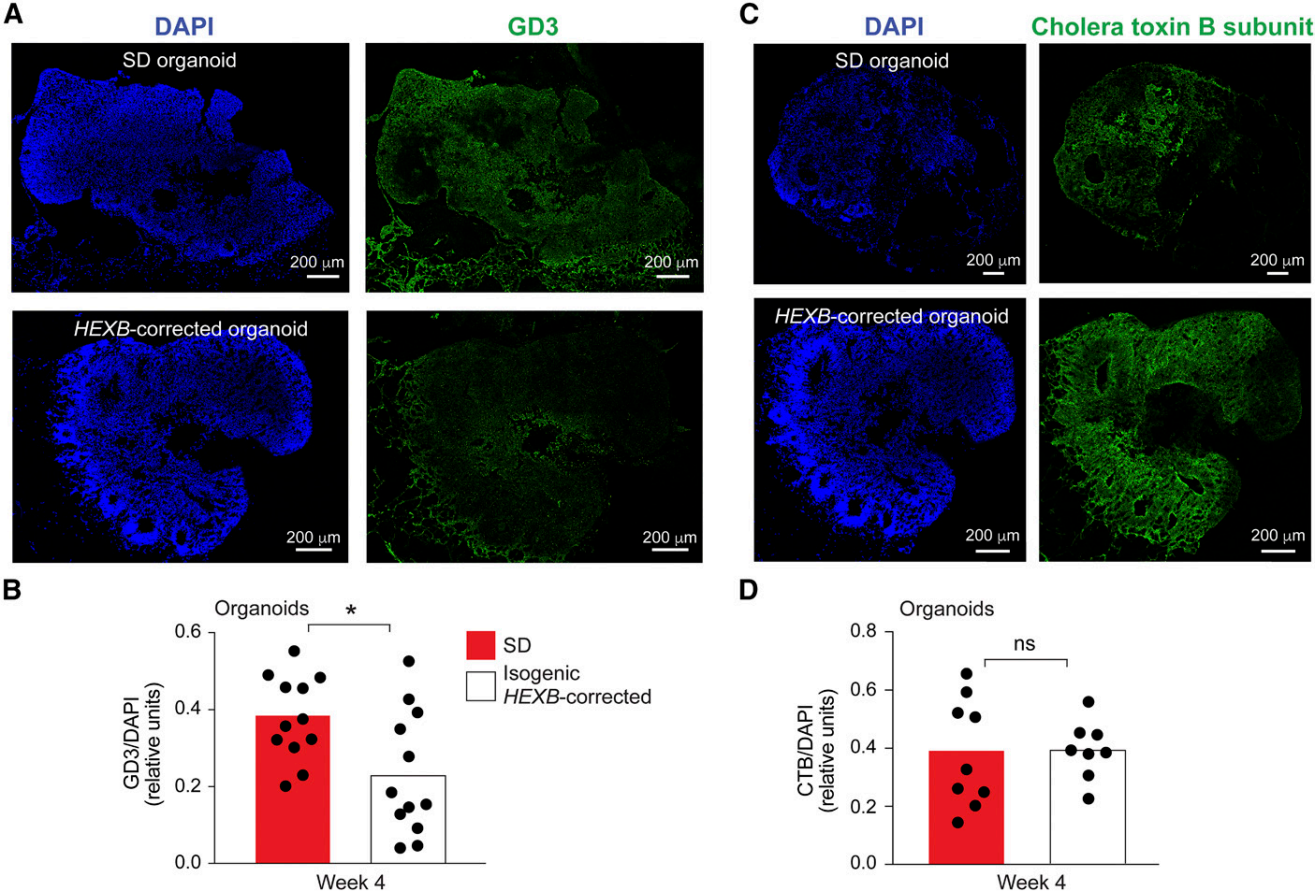
Expression of GD3 and GM1 in Sandhoff disease cerebral organoids. A: Frozen sections of Sandhoff disease (SD; top panels) and isogenic *HEXB*-corrected organoids (bottom panels) at week 4 of culture were stained with DAPI (left) and anti-GD3 ganglioside (right). Representative images of entire organoid sections are shown. B: Quantification of GD3 ganglioside expression in SD organoids (red bars) and isogenic *HEXB*-corrected organoids (white bars) at week 4 of culture. GD3 ganglioside expression was calculated as anti-GD3 ganglioside antibody fluorescence intensity normalized by DAPI intensity quantified by ImageJ software. Bars represent mean GD3 expression and each dot represents the value corresponding to an entire organoid section, with two sections per organoid. *P < 0.05, *t*-test analysis between SD and the corrected clone. C: Sections of Sandhoff disease (SD; top panels) and isogenic *HEXB*-corrected organoids (bottom panels) at week 4 of culture were stained with DAPI (left) and FITC-conjugated cholera toxin B subunit (CTB) (right). Representative images of entire organoid sections are shown. D: Quantification of CTB binding in SD organoids (red bars) and isogenic *HEXB*-corrected organoids (white bars) at week 4 of culture. CTB binding was calculated as CTB fluorescence intensity normalized by DAPI intensity quantified by ImageJ software. Bars represent mean CTB binding and each dot represents the value corresponding to an entire organoid section, with two sections per organoid. ns, not significant; *t*-test analysis between SD and the corrected clone.

We measured the levels of GD3 ganglioside, abundantly expressed on developing neurons (30). GD3 ganglioside expression, detected by immunostaining, was significantly reduced in the 4 week isogenic *HEXB*-corrected organoids compared with Sandhoff organoids (**Fig. 4A, B**). Cholera toxin B subunit binding, which measures GM1 ganglioside and other related glycolipids (31), was not significantly different between Sandhoff disease and *HEXB*-corrected organoids at 4 weeks (Fig. 4C, D).

**Fig. 5.**
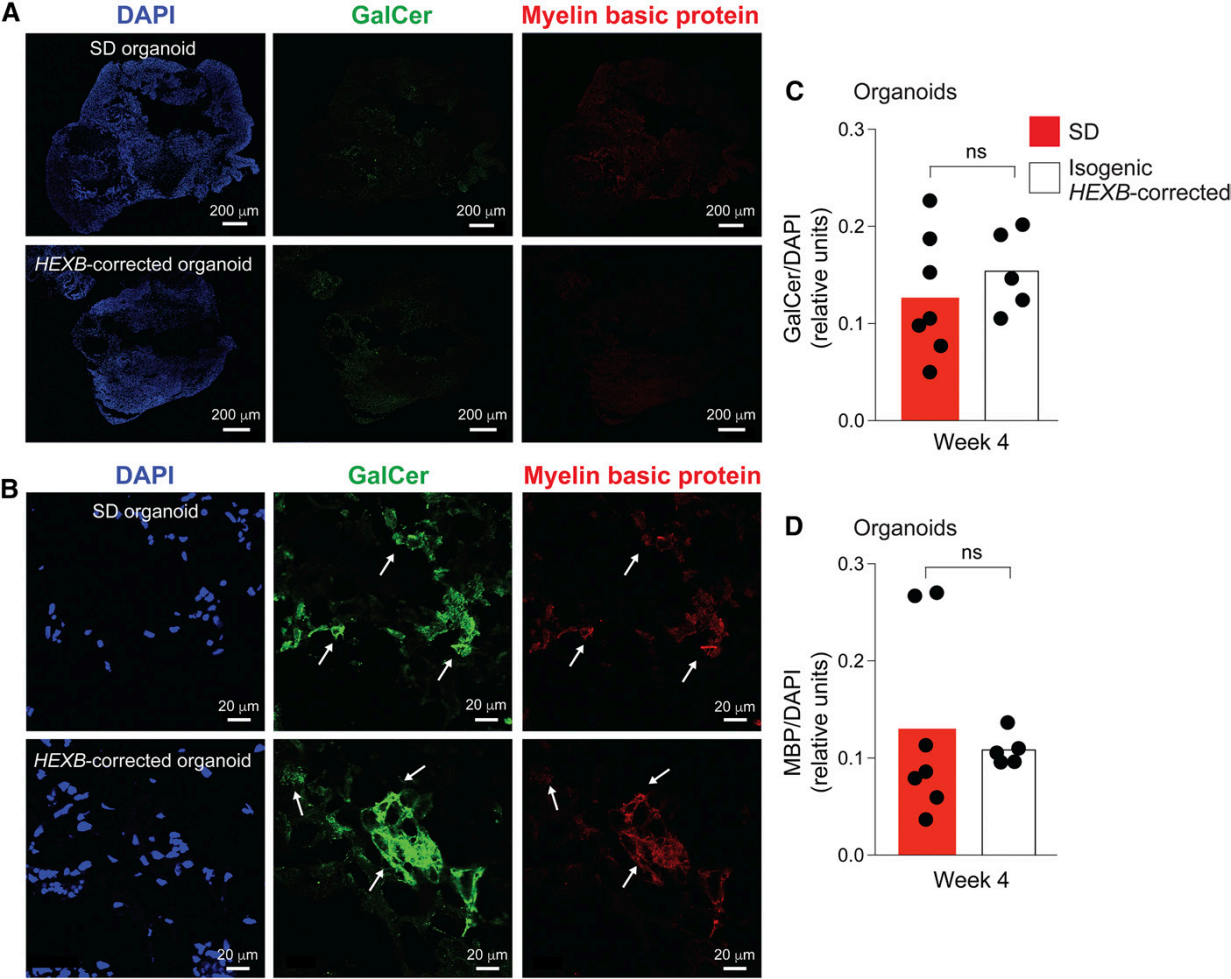
Expression of galactosylceramide and myelin basic protein in Sandhoff disease cerebral organoids. A, B: Frozen sections of Sandhoff disease (SD; top panels) and isogenic *HEXB*-corrected organoids (bottom panels) at week 4 of culture were stained with DAPI (left), anti-galactosylceramide (GalCer) (center), and myelin basic protein (MBP) (right). Representative images of entire organoid sections are shown in A and higher magnification views in B. C, D: Quantification of GalCer and MBP expression in SD organoids (red bars) and isogenic *HEXB*-corrected organoids (white bars) at week 4 of culture. GalCer and MBP expression was calculated as antibody fluorescence intensities normalized by DAPI intensity quantified by ImageJ software. Bars represent mean GalCer and MBP expression and each dot represents the value corresponding to an entire organoid section, with two sections per organoid. ns, not significant, *t*-test analysis between SD and the corrected clone at each time point.

Galactosylceramide, a major glycosphingolipid of myelin (32), was probed by immunostaining 10 week Sandhoff and isogenic *HEXB*-corrected organoid frozen sections (**Fig. 5A, C**). Expression of galactosylceramide, which was not significantly different between Sandhoff and isogenic *HEXB*-corrected organoids, was largely coexpressed with an oligodendrocyte marker, myelin basic protein (Fig. 5B, D). The results indicate that galactosylceramide production was not disturbed in the Sandhoff disease organoids.

### Sandhoff disease cerebral organoids are enlarged and exhibit increased cellular proliferation

**Fig. 6.**
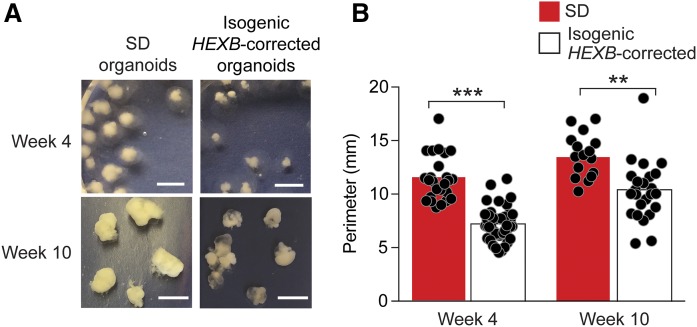
Sandhoff disease cerebral organoids are larger than isogenic *HEXB*-corrected cerebral organoids. A: Representative images of the cerebral organoids at weeks 4 and 10 of culture. Scale bar, 0.5 cm. B: Size comparison of Sandhoff disease (SD; red bars) and isogenic *HEXB*-corrected organoids (white bars) at week 4 and week 10 of culture performed by calculating the perimeter of each organoid using ImageJ software. The bars represent mean perimeter values and each dot represents one organoid. **P < 0.01, ***P < 0.001, *t*-test analysis between SD and corrected organoids.

When the size of the organoids was examined, the Sandhoff disease organoids appeared larger than the organoids generated from the isogenic *HEXB*-corrected cells at weeks 4 and 10 of culture (**Fig. 6A**). We then calculated the size of individual cerebral organoids (11, 12, 23, 27, 28). This analysis showed that Sandhoff disease organoids were significantly larger in size compared with the isogenic *HEXB*-corrected organoids at week 4 and week 10 of culture (Fig. 6B).

**Fig. 7.**
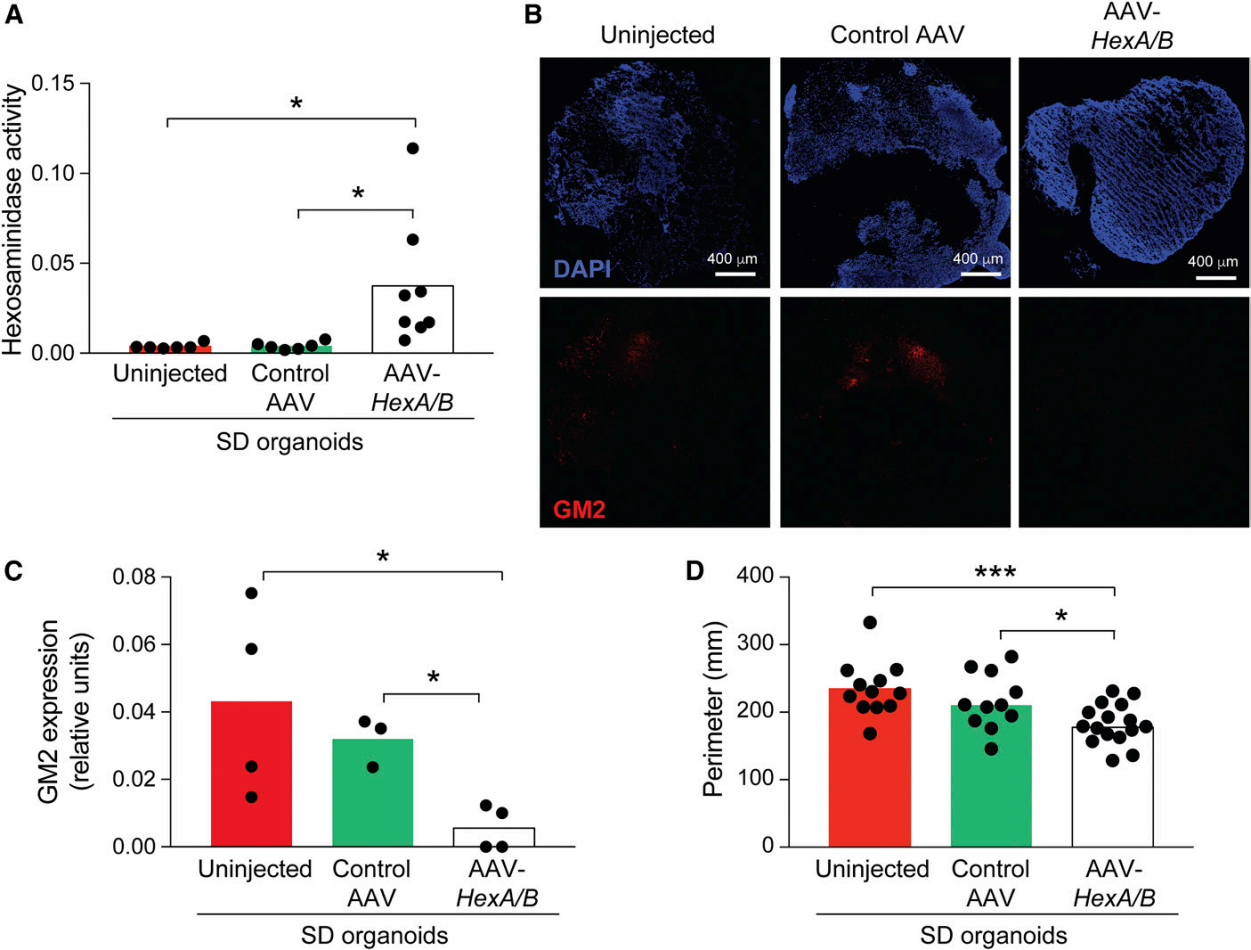
AAV mediated β-hexosaminidase correction reduced GM2 storage and size of Sandhoff disease cerebral organoids. A: β-hexosaminidase activity of uninjected, control AAV-GFP-injected, and AAV*-HEXA/B*-injected Sandhoff disease cerebral organoids. The bars represent average β-hexosaminidase activity normalized by β-galactosidase activity per minute and each circle represents one individual organoid. *P < 0.05, *t*-test. B, C: Expression of GM2 in AAV-injected Sandhoff disease organoids. Representative images of entire organoid sections 11 days after injection of control AAV-GFP-injected (central panels), AAV*-HEXA/B-*injected (right panels), and uninjected organoids (left panels). GM2 ganglioside expression, bottom panels; DAPI staining, top panels. C: Quantification of GM2 ganglioside expression. The bars represent average GM2 ganglioside fluorescence intensity normalized by DAPI intensity and each circle represents one individual organoid. *P < 0.05, *t*-test. D: Quantification of organoid size as described in Fig. 6. The bars represent perimeter average values and each circle represents one individual organoid. *P < 0.05, ***P < 0.001, *t*-test.

To demonstrate that the β-hexosaminidase deficiency was responsible for the increased size of the Sandhoff disease organoids, we injected the organoids with AAV carrying monkey *HEXA* and *HEXB* genes (AAV*-HEXA/B*) to restore expression of β-hexosaminidase. The organoids injected with AAV*-HEXA/B* showed significantly increased β-hexosaminidase activity, significantly less GM2 storage, and reduced size compared with organoids injected with a control virus (AAV-GFP) or uninjected organoids (**Fig. 7A–D**).

**Fig. 8.**
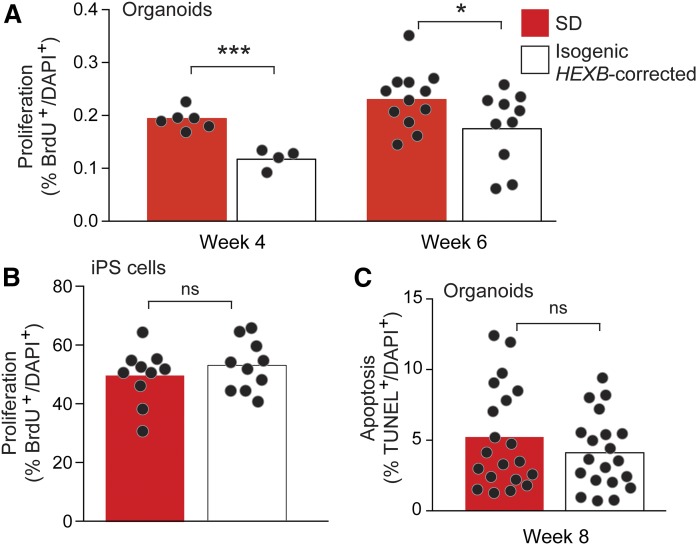
Sandhoff disease cerebral organoids display increased proliferation. A: Proliferation of cells in organoids. Quantification of the percentages of BrdU^+^ DAPI^+^ cells in Sandhoff disease (SD; red bars) and *HEXB*-corrected organoids (white bars) at week 4 and week 6 of culture. The bars represent mean values corresponding to random fields (dots) taken from entire organoid sections (three organoids for SD and two organoids for *HEXB*-corrected at 4 weeks; six organoids for SD and four organoids for *HEXB*-corrected at 6 weeks). B: Proliferation of iPS cells. Quantification of the percentages of EdU^+^ DAPI^+^ SD and *HEXB*-corrected iPS cells. Ten random fields (dots) were counted per cell type. The bars represent mean values. C: Apoptosis of cells in organoids. SD and *HEXB*-corrected organoids at week 8. Quantification of the percentages of TUNEL^+^ DAPI^+^ cells. Ten random fields (dots) were counted per genotype. The bars represent mean values. Two organoids were analyzed for each genotype. *P < 0.05, ***P < 0.001, *t*-test analysis between SD and each corrected clone. ns, not significant.

To determine the basis of the size differential between Sandhoff disease and isogenic *HEXB*-corrected organoids, we examined the proliferation index in organoids by analyzing cellular BrdU incorporation at week 4 and week 6 of culture. We found a significant increase in the number of BrdU^+^ DAPI^+^ cells in Sandhoff disease organoids compared with the isogenic *HEXB*-corrected organoids at both time points (**Fig. 8A**). However, no significant differences were found in the proliferation capacity of the Sandhoff disease and isogenic *HEXB*-corrected iPS cells (Fig. 8B), indicating that the difference in cellular proliferation was a property of the more differentiated organoids. We detected low percentages of TUNEL^+^ DAPI^+^ apoptotic cells in Sandhoff disease organoids that were not significantly different from the *HEXB*-corrected organoids at week 8 (Fig. 8C), suggesting that cell death was not a factor in the difference in organoid size.

### Sandhoff disease cerebral organoids exhibit impaired neuronal differentiation

**Fig. 9.**
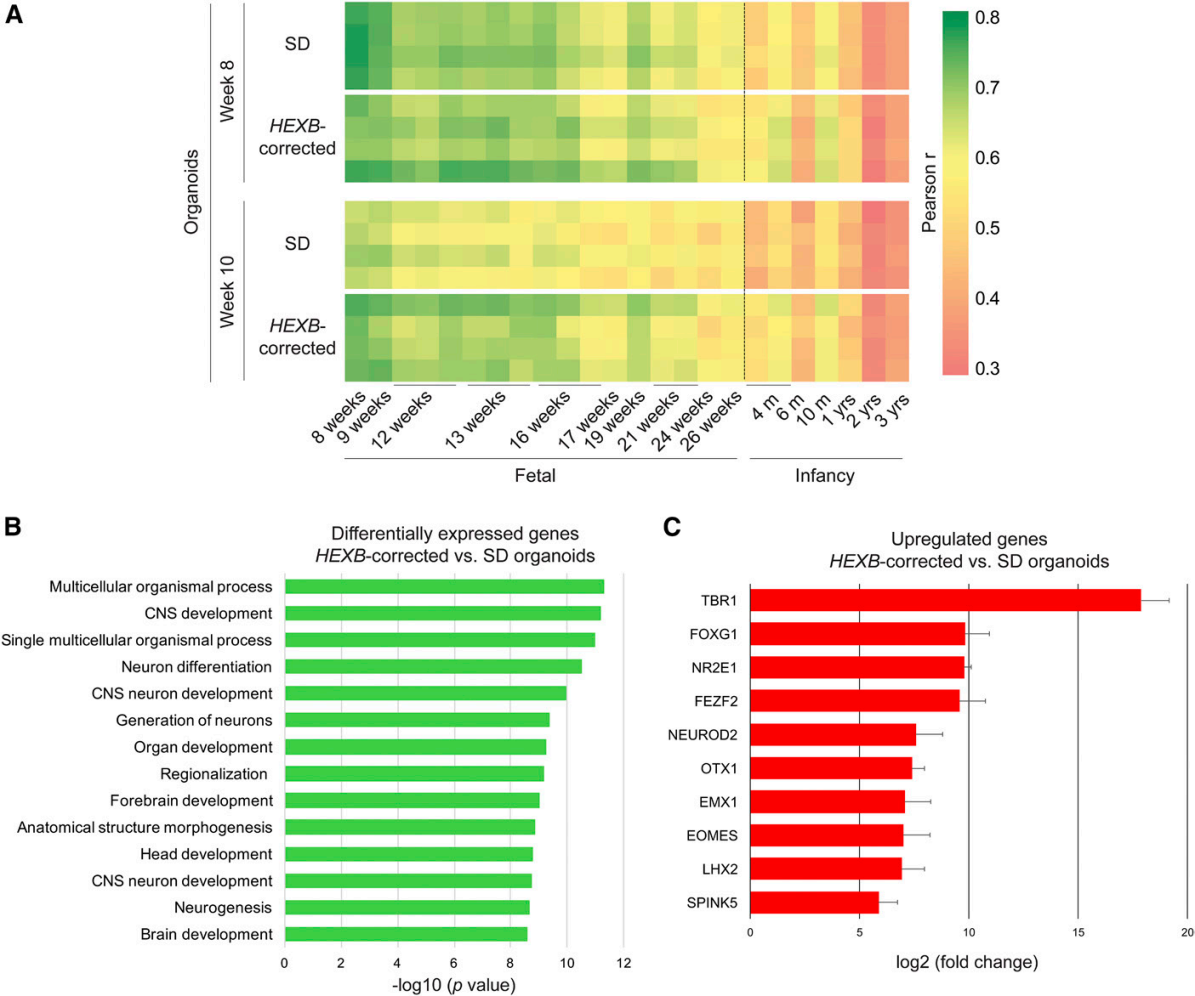
Sandhoff disease cerebral organoids exhibit dysregulated expression of genes related to CNS development. A: Heat map of Pearson correlation analysis of RNA-Seq data for four Sandhoff disease (SD) and four isogenic *HEXB*-corrected individual organoids with a published transcriptome database of human dorsolateral prefrontal cortex across two developmental stages. B: Gene ontology analysis of the top 100 upregulated and top 100 downregulated genes that are differentially expressed in *HEXB*-corrected organoids compared with SD organoids at week 10 of culture. Biological processes category was plotted selecting the top 14 terms ranked by *P* value. C: Top 10 significantly upregulated genes in *HEXB*-corrected organoids compared with SD organoids at week 10 of culture, shown as fold change of the gene expression (RPKM).

We next performed whole-transcriptome RNA-Seq analysis to determine whether developmental differences were reflected in gene expression profiles of Sandhoff disease and isogenic *HEXB*-corrected cerebral organoids. First, we identified the developmental stage of the cerebral organoids by comparing the organoid gene expression profiles with expression data from 16 human dorsolateral prefrontal cortex samples corresponding to different fetal and infancy stages (33). Pearson correlation analysis (23, 24) indicated that the gene expression profiles of Sandhoff disease and isogenic *HEXB*-corrected organoids at 8 weeks correlated most highly with those corresponding to fetal human dorsolateral prefrontal cortex samples from 8 to 16 weeks of pregnancy (**Fig. 9A**, top panel). At week 10 of culture, there was a divergence between the Sandhoff disease and isogenic *HEXB*-corrected organoids in their correlation with the fetal brain samples, suggesting differences in their gene expression profiles (Fig. 9A, bottom panel).

Gene ontology analysis of the top 100 upregulated and top 100 downregulated genes for *HEXB*-corrected organoids compared with Sandhoff disease organoids revealed that the top biological process pathways identified were predominantly associated with central nervous system and neuronal differentiation (Fig. 9B). Moreover, the top nine upregulated genes in isogenic *HEXB*-corrected organoids relative to Sandhoff disease organoids encoded transcription factors critical in neuron morphogenesis and central nervous system development: *TRB1* (34), *FOXG1* (35), *NR2E1* (36), *FEZF2* (37), *NEUROD2* (38), *OTX1* (39), *EMX1* (40), *EOMES* (41), and *LHX2* (42) (Fig. 9C). The elevated expression of these genes indicated that neuronal differentiation was more advanced in isogenic *HEXB*-corrected organoids than in Sandhoff disease organoids.

## DISCUSSION

The impact of lysosomal storage diseases on neurodevelopment has been challenging to determine. Through the confluence of iPS cell, CRISPR/Cas9, and organoid technologies, it is now possible to study the brain-specific pathophysiology of human disease in an in vivo setting (13, 14, 43). Here, we describe an analysis of infantile Sandhoff disease using human cerebral organoids generated from patient-derived iPS cells. To isolate the effect of the disease-causing mutations in the *HEXB* gene from the patient-specific genetic background, we generated isogenic *HEXB*-corrected iPS cells using CRISPR/Cas9 genome editing to serve as controls in our studies. A simple screen, based on the enzymatic detection of recovered β-hexosaminidase activity, was devised that facilitated identification of correctly edited Sandhoff disease iPS cells, which were confirmed by sequencing. This enzymatic activity-based screening procedure may be a useful adjunct for the genetic editing of iPS cells for other lysosomal storage or metabolic diseases in which enzyme deficiencies are present and easily determined.

The three-dimensional organoid model that was utilized mimicked neuronal differentiation processes occurring during fetal development in human cortex (12, 23, 24, 44, 45). Cerebral organoids have been used previously for modeling normal and pathological development in a variety of contexts, including microcephaly, autism, and Alzheimer’s disease (12, 27, 46). The Sandhoff disease cerebral organoids generated in this report stored increased amounts of GM2 ganglioside beginning at 4 weeks, which progressively increased with time in culture. Gene expression profiling suggested that the organoids at 8 weeks of culture were developmentally similar to 8–16 weeks postconception human fetal cerebral cortex. Lipid storage in the brains of fetuses affected with GM2 gangliosidosis has been noted as early as the first trimester of pregnancy (47–51), in accordance with the results obtained here with cerebral organoids.

Cerebral organoids have been shown to mimic clinical conditions of abnormal head size in infancy and childhood, including microcephaly, in patients with *CDK5RAP2* mutations (12) and Zika infection (23) and megacephaly, in autism spectrum disorders (27). The Sandhoff disease patient from whom the iPS cells were derived for this study exhibited megalencephaly, a general feature of GM2 gangliosidosis patients. The Sandhoff disease organoids were significantly larger in size than the isogenic *HEXB*-corrected organoids and the Sandhoff disease organoids injected with AAV-*HEXA/B* evoking a megalencephalic-like condition. It is unclear whether the abnormally increased cellular proliferation detected in the Sandhoff disease organoids substantially contributes to the GM2 gangliosidosis-associated megalencephaly in patients; however, if the proliferative defect extended into the postnatal period, it could be clinically impactful. Further studies will be required to clarify whether there is a causal relationship between the increased proliferation within the Sandhoff organoids and the clinical finding of megalencephaly in the GM2 gangliosidoses.

Transcript profiling indicated that pathways involved in neuronal differentiation and central nervous system development were altered in the cerebral organoids derived from the Sandhoff disease iPS cells compared with those generated from the isogenic *HEXB*-corrected controls. Strikingly, correction of the *HEXB* mutation and restoration of β-hexosaminidase activity upregulated several key transcription factor genes involved in neuron morphogenesis and central nervous system development. These findings suggest that neuronal differentiation is impeded in Sandhoff disease, and are in accordance with work in the Sandhoff disease mouse model that showed progenitor cells with lysosomal β-hexosaminidase deficiency exhibited impaired neuronal differentiation (9, 10). The elevated expression of GD3 ganglioside observed in the Sandhoff disease organoids is also consistent with reduced neuronal maturation (30).

How the lysosomal β-hexosaminidase deficiency causes abnormal cellular proliferation and neuronal differentiation of cerebral organoids is not known. However, glycosphingolipids, which are substantially increased in the GM2 gangliosidoses, are known to influence growth and differentiation of cells in a variety of experimental contexts (32, 52–55). The accrual of gangliosides can directly promote neural stem cell proliferation (56–58) and has been implicated in neuronal differentiation (30, 59–63). A variety of mechanisms have been described for the influence of gangliosides on cell behaviors, including direct modulation of signaling receptors and mediation of cell-cell interactions (63–68). Interestingly, lysosomal storage in cells from a related disorder, GM1 gangliosidosis, activates mTORC1 signaling (69), which is a pathway directly linked to cellular proliferation. However, we cannot rule out the possibility that a substrate of β-hexosamindase other than GM2 ganglioside (Fig. 1A) may influence cellular proliferation and differentiation within cerebral organoids.

Early neurodevelopmental processes are believed to have little impact on the major acute symptoms of Sandhoff disease (8). This conclusion was reached using an inducible adult mouse model of *Hexb* deficiency whose disease severity was indistinguishable from a germline deficiency (8). Indeed, within the Sandhoff disease organoids, we did not observe an elevation of apoptosis, which is a major factor in the terminal neurodegenerative disease course. However, the results revealing an altered proliferation and neuronal differentiation status exhibited by the Sandhoff disease organoids raise the possibility of more subtle neurodevelopmental abnormalities that might be masked by the severe stereotypical features of the GM2 gangliosidoses.

The three-dimensional cerebral organoid model of Sandhoff disease provides a new means to study the early developmental consequences of lysosomal ganglioside storage within a human context. Using this paradigm, alterations in fundamental cellular behaviors, proliferation and neuronal differentiation, were identified, which raise the possibility that they could impact fetal brain development in GM2 gangliosidosis patients. While perhaps not contributing to acute neurodegeneration, they may be consequential when treatments are eventually devised to reverse enzyme deficiencies postnatally (70–72).

## Supplementary Material

Supplemental Data
