# Supplementary material for: Cerebral organoids derived from Sandhoff disease-induced pluripotent stem cells exhibit impaired neurodifferentiation
Source: J Lipid Res. 2018 Jan 22;59(3):550–63. doi: 10.1194/jlr.M081323 (PMC5832932; doi:10.1194/jlr.M081323)
Supplement: Supplemental Data [file supp_59_3_550__index.html]

Cerebral organoids derived from Sandhoff disease induced pluripotent stem cells exhibit impaired neurodifferentiation — Cerebral organoids derived from Sandhoff disease-induced pluripotent stem cells exhibit impaired neurodifferentiation — Supplemental Data 

# Cerebral organoids derived from Sandhoff disease-induced pluripotent stem cells exhibit impaired neurodifferentiation

## Supplemental Data

- Supplemental table S1 (.docx, 33 KB) - Primers used for sequencing of the predicted off-target loci of the HEXB-targeted sgRNA
- Supplemental table S1 (.docx, 34 KB) - Sequence analysis of the predicted off-target loci of the HEXB-targeted sgRNA in the HEXB-corrected iPS cell clone.
