# Supplementary material for: Cerebral organoids derived from Sandhoff disease-induced pluripotent stem cells exhibit impaired neurodifferentiation
Source: J Lipid Res. 2018 Jan 22;59(3):550–63. doi: 10.1194/jlr.M081323 (PMC5832932; doi:10.1194/jlr.M081323)
Supplement: Supplemental Data [file 10.1194_M081323_jlr.M081323-1.docx]

**Supplemental Table S1**. Primers used for sequencing of the predicted off-target loci of the *HEXB*-targeted sgRNA

| Off-target | Forward primer | Reverse primer | Fragment size |
| --- | --- | --- | --- |
| 1 | 5' AACAGCACAAGTAGCACTTTG 3' | 5' ACAGAACGAAAAGGGAGCTAA 3' | 165 bp |
| 2 | 5' CCTCTGTCCTATTTTGAGAGT 3' | 5' GTGCAATTAGAGGTAAAGAATGA 3' | 189 bp |
| 3 | 5' AAACCCAGGGAGAGTCATAAG 3' | 5' CCAAAAACACTCTTCTGTGGA 3' | 177 bp |
| 4 | 5' CACACAACTTTCTAAAGTTCC 3' | 5' GAGTCCTTCCAAAAGAACTTG 3' | 203 bp |
| 5 | 5' TTGACCAGGAAGACAGACAAA 3' | 5' TCAAGCATGCTTGTAATTTGG 3' | 197 bp |

Potential off-target genomic DNA sequences for the sgRNA were predicted by the Optimized CRISPR Design Web site (15). The top five off-target loci were sequenced.
