# Supplementary material for: Cerebral organoids derived from Sandhoff disease-induced pluripotent stem cells exhibit impaired neurodifferentiation
Source: J Lipid Res. 2018 Jan 22;59(3):550–63. doi: 10.1194/jlr.M081323 (PMC5832932; doi:10.1194/jlr.M081323)
Supplement: Supplemental Data [file 10.1194_M081323_jlr.M081323-2.docx]

**Supplemental Table S2.** Sequence analysis of the predicted off-target loci of the *HEXB*-targeted sgRNA in the *HEXB*-corrected iPS cell clone.

| Off-target | Genomic location | Locus details | Sequence | #mm | Edited |
| --- | --- | --- | --- | --- | --- |
|  | HEXB sgRNA |  | gtaacgttaatggcttgcgc |  |  |
| 1 | chrX:-125791194 | Intergenic | GTTAAGATACTGGCTTGCGCTAG | 4 | no |
| 2 | chr5:-130028750 | Intergenic | GTAAAGTTAATGGCTTGAGACAG | 3 | no |
| 3 | chr3:-31540915 | Intergenic | GTTACGTCAATGGCTTGCTTTAG | 4 | no |
| 4 | chr1:-8757809 | Intergenic | GTAAAGTGAATGGCTTGCATAAG | 4 | no |
| 5 | chr2:-200083696 | Intergenic | GTAATGTAAATGACTTGCCCAAG | 4 | no |

Potential off-target genomic DNA sequences for the sgRNA were predicted by the Optimized CRISPR Design Web site (15). The top five off-target loci were sequenced. Mismatches are in red text; putative PAM sequences are highlighted in green. #mm, number of mismatches.
